# Hands-on-ground in a new country: a community-based participatory evaluation with immigrant communities in Southern Alberta

**DOI:** 10.1177/17579759231176293

**Published:** 2023-06-13

**Authors:** Ulises Charles-Rodriguez, Aiat Aborawi, Kamal Khatiwada, Ashmita Shahi, Silvia Koso, Savanna Prociw, Christa Sanford, Richard Larouche

**Affiliations:** 1 Faculty of Health Sciences, University of Lethbridge, Canada; 2 Faculty of Social Work, University of Calgary, Canada; 3 Community researchers, Lethbridge Family Services, Canada

**Keywords:** immigrants, refugees, health promotion, community gardens, participatory evaluation, community-based research/participatory research

## Abstract

Immigrants experience a high risk of mental health deterioration following settlement in Canada. Immigrant communities benefit from health-promoting interventions that stimulate social inclusion and belonging as protective factors. In this context, community gardens have been recognized as interventions that promote healthy behaviours, place attachment and belonging.

This article summarizes our experience conducting a community-based participatory evaluation (CBPE), engaging community stakeholders in planning, implementing and evaluating a community garden for immigrants. We conducted a CBPE to provide relevant and timely feedback to inform programme adaptation and development. Participants, interpreters and organizers were engaged through surveys, focus groups and semi-structured interviews. Participants expressed a range of motivations, benefits, challenges and recommendations. The garden was a place that fostered learning and promoted healthy behaviours, including physical activity and socialization. However, there were challenges in organization and communication with participants. Findings were used to adapt the activities to immigrants’ needs and expand the programming of collaborating organizations. Stakeholder engagement facilitated capacity building and direct use of findings. This approach may catalyse sustainable community action with immigrant communities.

## Introduction

Evidence suggests a five-fold increase in emotional and mental health problems among newcomers after six months of arrival in Canada (1). Moreover, refugees and immigrants with settlement problems present a higher risk of mental health deterioration (2). In Canada, migration is considered a social determinant of health (3) and a central focus of health promotion practice (4). As the world enters a new era of demographic flow, receiving societies must implement interventions that facilitate settlement (5,6).

Based on the strengthening community action strategy of the Ottawa Charter for Health Promotion, it is crucial to collaborate with immigrant service providers, grassroots organizations and leaders to maximize stakeholder engagement in sometimes hard-to-reach communities, such as newcomers, refugees and ethnic minorities (4,7). Additionally, collaborating with communities can strengthen health research’s relevance, quality and use (8). This article summarizes our experience conducting a community-based participatory evaluation (CBPE), engaging community stakeholders in planning, implementing and evaluating a community garden for immigrants in the city of Lethbridge, Alberta.

Previous research suggests that natural environments, including community gardens, can promote immigrants’ integration, well-being and physical activity (9). Immigrants who participate in community gardens have shown greater place attachment to their host country (10) and a greater sense of belonging (11–14). Some suggest that this is due to embedded socialization (12,15,16), while others propose that embodied experiences contribute to connecting memories (17), which help to establish meaningful connections with their new country (11,18). Regardless of the pathway, previous experiences suggest that community gardening can facilitate settlement (19), particularly among refugees (10).

Community gardens have been suggested approaches to promote healthy behaviours (15,20), particularly in those with agricultural backgrounds (11,16). Active gardeners have shown increases in vegetable and fruit intake (21–24) and better control of their diabetes (22). Baker (20) identified that immigrants tend to plant culturally relevant species, often not available in grocery stores, which speaks to their increased capacity to develop culturally appropriate food security. Participation in gardening can also be a protective factor against dietary acculturation (25).

Other studies suggest that gardening can be a source of physical activity (15,21,22,24), particularly among older immigrants (26). In immigrant populations, gardening can be a meaningful occupation (12) and an activity that enables self-actualization (18), and improves general well-being (15) and quality of life (19). Newcomers with agricultural backgrounds can practise previous knowledge and skills (17,27), which can translate to confidence that can permeate to other areas of life (13).

However, immigrant communities may experience multiple barriers when establishing community gardens, including access to resources (13,21,23), land (14–16) and the knowledge or connections required to navigate local rules and regulations. Previous studies have suggested that transportation (20,24,25), language (15,18,20) and opportunities for socialization (18) affect the level and quality of participation. Furthermore, engagement of immigrants in the design, implementation and evaluation of nature-based interventions has been minimal (9). Meaningful engagement with immigrant communities can help develop initiatives that address their specific needs and preferences.

Evaluations of immigrant community gardens have measured different health outcomes (21,23,28). Although these types of evaluations can be valuable to corroborate the effectiveness of interventions, other types of evaluations can be more instrumental in programme development. According to Patton (29), developmental evaluation is ideal for studying grassroots initiatives that start from local contexts and needs. Evaluators engage in collaborative experiments and community development, adapting initiatives to changing realities and regional specificities. The evaluation design focused on the potential use of findings, providing feedback for development. We decided to conduct a CBPE engaging immigrants in all project stages to strengthen the community and build capacity for sustainable action (30).

## Methods

### Setting and context

The city of Lethbridge is located in Alberta, east of the Canadian Rocky Mountains and about 100 km north of the US border. The 2021 census calculated a population of 98,406, with an immigrant population of 14,485 (31). International migration to the city has increased, from a rate of 1,100–1,200 per decade in the 1980s and 1990s, to nearly 6,000 in the 2010s (31), making migration a major driver of Lethbridge’s demographic growth.

During the last 10 years, the city of Lethbridge has been the setting of two community gardens for newcomers. Anecdotal accounts of both initiatives indicate that participants were highly receptive. Despite this, communities faced significant challenges leading, organizing, maintaining, adapting and sustaining their projects. Most importantly, they have depended on organizations that do not prioritize their interests. When these organizations decided to stop lending their land or when grants and funding to support community gardening initiatives ran out, immigrant communities could not find new places for gardening. Accessing land and creating a community garden requires both financial and social capital, which is in early development in the case of newcomers.

### Initial steps in developing partnerships

In the fall of 2020, the lead author mapped the city’s immigrant community ecosystem (7) and contacted stakeholders from different sectors, including a settlement programme, a local food bank, grassroots associations, a local church and local immigrant leaders. The network held a series of meetings where stakeholders discussed previous community garden experiences and possible solutions that could grant gardening space to newcomers and immigrant communities.

### Action

The local food bank offered its community garden as a location for immigrant groups to collaborate in planting and maintaining activities for the 2021 gardening season. Agencies and community leaders organized volunteer groups in compliance with the maximum outdoor gathering capacity allowed during the COVID-19 pandemic. More than 20 immigrants participated either independently or as part of a group. Participants from the settlement programme were provided with interpretation services.

### Evaluation methods

Two guiding questions set the direction of the evaluation: how was the experience of immigrants participating in the volunteer programme at the Interfaith Learning Garden? And what was the best possible direction for the following gardening season? The evaluation included five focus groups and three individual semi-structured interviews with key informants. Both interviews and focus groups were semi-structured, using probing questions focusing on participants’ experiences and recommendations for future programming. Participants completed a questionnaire about their demographic information, previous gardening experience and satisfaction levels with the programme and garden operations. Interviews of 20–60 min and focus groups were conducted in participants’ native languages with interpretation services. The research assistant, research collaborators and lead author transcribed audio-recorded interviews, focus groups and notes to Microsoft Word documents and translated them to English. Focus groups, interviews and questionnaires were triangulated to detect inconsistencies and strengthen the analysis. The principal investigator and the research assistant performed qualitative content analysis (32) using an initial coding frame based on probing questions. They independently performed a trial coding using the NVivo 1.6.1 software, then evaluated and modified the coding frame until consensus was reached in a data matrix.

We used Chouinard and Cousins’s (33) three dimensions of participatory practice to maximize participation in every stage of the evaluation:

*Diversity among stakeholders*: Three members of the settlement programme and two members from the local food bank collaborated on the evaluation. One undergraduate student was hired as a research assistant, and two graduate students participated as collaborators in the implementation and evaluation of the project – all are from local immigrant communities. The team included an evaluation specialist, a public health researcher, and the lead author – an immigrant.

*Ownership of the evaluation process*: Stakeholders from the settlement programme, the local food bank and student collaborators were part of the decision-making processes for the design, implementation and evaluation of the intervention.

*Extent of participation*: The depth of participation resulted from stakeholders’ interest and capacity. The evaluation specialist, the senior researcher, the lead author, one research collaborator and representatives from the settlement programme and the food bank participated in selecting the study design, methods and probing questions. One member of the settlement programme, the research assistant, the lead author and the two research collaborators collected the data. The lead author and the research assistant performed the data analysis.

## Findings

Fourteen participants engaged in focus groups, and five key informants were interviewed, including one participant, two interpreters and two organizers. Most focus groups and interviews were conducted at the food bank, except one focus group conducted at an ethnic organization and one interview conducted at a participant’s chosen location. Participation was voluntary and not remunerated. Most focus groups took place before cooking sessions, where food and beverages were provided. Six participants were settled immigrants (with more than one year in Canada, fluent in English and working) and eight were newcomers (participating in the settlement programme). Participants were from seven different nationalities, namely Bhutan, Mexico, Sudan, India, Congo, Eritrea and Pakistan. The average age was 45 years old and nearly three-quarters self-identified as females. Seventy-five percent of participants had gardened in their country of origin, 58% in Canada and 27% in transition countries. One author participated in the garden as a volunteer but was not involved in any type of data collection. Five main themes emerged from the data and are discussed in the following sections along with the participants’ recommendations.

### Themes

We structured our findings in five themes and two subthemes: 1) motivation, with learning and health benefits as subthemes; 2) positive outcomes; 3) challenges; 4) other experiences; and 5) participants’ recommendations.

1 Motivation
a) Learning

Learning was the most common motivation for both newcomers and settled immigrants. Many expressed their interest in learning from seed to harvest, including soil enrichment, appropriate care, and seeding and harvest times. Some were encouraged to participate because gardening was an activity they missed from home. One participant shared:

Even some of the names, we don’t know them. We knew them back home when we were doing gardening in our house, but when we came here, we didn’t know [the names] or where to get the seeds. So, when I came here to the garden, I learned a lot from it: how to do it [gardening], where I can find seeds, and a lot of information for us. (MS)

In addition, many participants expressed their interest in getting to know Canadian gardening conditions, including weather, soil conditions, local practices and techniques, and native plants. A few participants stated that their interest was to apply what they learned at home so they could create or improve their gardens. Some participants were interested in teaching their children to garden. For those with agricultural backgrounds, it was a way to preserve traditions and transmit skills and knowledge. Only one participant expressed interest in learning large-scale techniques that could lead to entrepreneurial activities.

b) Health benefits

Different health benefits were mentioned as motivators during the focus groups, particularly socialization, having access to fresh vegetables for their own consumption, and physical activity. For participants from the Bhutanese community, exercise was one of their primary motivations; they contrasted sedentary lifestyles with being active and supporting the community. One of them noted:

We go to exercise. It is boring to stay at home. When we go [to the community garden], it is beneficial for our body; at the same time, we get to help and support the community. (BM)

For some participants who did not belong to any previously established community, meeting people was a motivation for participating. Unfortunately, there were minimal opportunities for out-of-group socialization, primarily due to the COVID-19 public health restrictions. The lack of socialization was associated with a loss of motivation and early withdrawal.

2. Positive outcomes

The average satisfaction with participating in the garden and specific activities performed was 6.5 on a 7-point scale. Gardening kept participants active and gave them ‘a break’ from daily activities and life demands. Participants with little or no gardening experience mentioned being excited when seeing plants grow, as one of them stated:

This thing that we only see them [strawberries] in the grocery store, and you see them in a package. So, seeing them there [in the garden] was like ‘so, that’s how they grow, that’s how they actually look like, wow!’ (AT)

Many participants expressed their satisfaction with having a space to socialize in the garden. One organizer stated that the feeling that the other people around ‘may be in the same boat as you’ probably facilitated socialization. One participant reflected on why a garden could be an ideal setting to meet people:

I truly think that [the garden] is a place where, if there are other people with the same disposition, conversations can happen because you are not doing a lot [laughs]. You are doing very monotonous activities so it’s very easy to start a conversation. (AT)

In the garden, participants engaged in various forms of communication, including hand gestures. One organizer mentioned that participants drifted to similar group languages, facilitating socialization even when struggling with English. The other organizer witnessed, by the end of the season, how participants with limited English did their best to communicate to express their gratitude.

Participants from the settlement group reportedly enjoyed the company of other women and learning from each other and their respective cultures, such as medicinal use of plants and recipes. For some participants, getting to know community resources was vital. Most participants mentioned having learned valuable tips about gardening in Canada. Others stressed that having exposure to Canadian gardening techniques made them aware that they preferred their traditional ways of farming. An African participant shared:

Back home in Africa, we don’t have those kinds of gardening, in pots. They have a bigger farm and you go farming . . . So, it gives me an idea of saying ‘even though I don’t have a farm, I can garden in a small place’. (EC)

3. Challenges

Newcomers experienced a significant number of challenges. The most frequently mentioned were language limitations and the need for interpretation services. Some participants and one organizer mentioned that relying on interpretation was very complicated. When a volunteer interpreter who also provided transportation stopped participating, it caused premature withdrawal of those who depended on him.

Most participants were willing to visit the garden more often. However, work and life schedules were mentioned as a barrier to increasing participation, especially when exploring the possibility of gardening more often. Some explained the difficulty of having to work most of the week or having busy parenting schedules. Others mentioned transportation as an essential barrier, particularly not knowing bus routes, not having a driver’s licence or a car, or not having someone to drive them (most often among seniors).

COVID-19 was a challenge for socialization. The gardening seasons started during strict public health restrictions, including the number of people in outdoor gatherings. As such, small-group visits and inter-group interaction was minimal. A participant expressed:

In that sense, I do feel that the objective was not fulfilled, in the sense of creating networks and meeting other people. (CA)

Outdoor gathering restrictions also prevented the settlement programme from referring other newcomers to the garden. The agency was forced to select participants using subjective assessments about who would benefit most and who would actually participate. In their experience, refugee women experience greater family caring obligations and barriers to socialization, therefore, the agency selected women who attended one of their life-skills enhancement groups. According to them, the process of elimination left out many who could have benefited from the programme.

Other participants expressed not having received sufficient information about the programme, particularly its food donation policy. Although participants were informed at the start that the garden’s main purpose was to contribute to the food donation programme of the food bank with fresh produce, their inability to access significant amounts of produce they have grown was a source of dissatisfaction.

The organizer from the settlement programme stated that having consistency in their groups tends to be difficult. The garden was an exception with high attendance rates. The organizer from the food bank had difficulties preparing group activities and scheduling equally engaging activities throughout the week. Both organizers shared the same concern but agreed that this was an excellent start. Participants with farming experience stressed that the space offered by the community garden was too small for the number of volunteers.

4. Other experiences

Both organizers stressed that the volunteers were on a spectrum of gardening knowledge and experience, which made it difficult to meet diverse expectations. Participants stated that they received clear instructions about the day-to-day activities, but they complained about not having well-rounded explanations about gardening processes (from seed to harvest). For experienced participants, this was ideal, but new gardeners would have preferred more explanations. A participant narrated:

We started to understand as time passed. The following week it was clear; we moved the soil. The week after, there were holes on the ground that we measured. So, we started to understand that it was a process but not because it was explained to us. (CA).

Volunteering in the food bank garden triggered different feelings about commitment and ownership. The lack of gardening independence (e.g. no participation in deciding what or when to plant and harvest) was experienced as a lack of ownership by some. However, others appreciated that the maintenance of the garden did not rely on them, but on the organizers. Many stressed that the garden was not theirs – neither the space nor its produce. The level of satisfaction with donating most of the vegetables to the food bank was one of the lowest graded items in the survey. Settled immigrants were satisfied with taking home symbolic amounts, whereas newcomers wanted to take greater amounts, as they were concerned about their food security.

5. Participants’ recommendations

The most common recommendation among participants was to expand gardening space, mostly by creating a new community garden for them. When exploring the possibility of creating a new garden, some participants recommended gardening alternatives, such as on-ground intensive farming or ethnic-specific spaces. Others highlighted the importance of keeping specific plots for food donation or stressed their concern regarding access to resources such as property and tools. Most participants acknowledged that having an independent garden would entail more work and organization. Many stated that they would be willing to increase their commitment if they had their own garden. However, most participants agreed that they needed guidance, ideally an expert who could teach them how to maintain the garden during and after the season. Several participants, particularly newcomers, expressed their need to have an organizer that would ‘keep them together’. Various participants expressed their desire to foster social relationships in the garden, including friends and family. A participant stated:

I understand the community part of it as an activity where we all ‘get our hands dirty’, we can all plant, and we can all harvest, but I think that it’s also important to reinstall the social interaction part of being in the community. (AT)

A frequent recommendation for the existing garden was to loosen the food bank take-home policies. One organizer stressed that some newcomers face food insecurity, and connecting them with the food donation programme or allowing them to take some produce would be meaningful for them. New gardeners suggested taking symbolic amounts that could reinforce their learning experience. Finally, a couple of newcomers mentioned they would greatly benefit from getting formal recognition for volunteering at the garden, which could facilitate their entry into the Canadian labour market.

### Continuing action

The research team submitted an evaluation report to collaborating agencies in April 2022, including recommendations. The lead author discussed with collaborating agencies to adapt programmes and activities to better suit participants’ needs and preferences. The food bank’s garden made significant modifications to its programme, focusing on the educational component, improving communication, and expanding events promoting socialization and community building. Other members of the community who were not active gardeners were also engaged – for example, a group of women participated in yoga sessions at the garden.

Additionally, the settlement programme and a local church have plans to create their community gardens, one serving newcomers and the other food-insecure refugee families. These projects will enable immigrants to enjoy gardening independence and food security outcomes while minimizing transportation and interpretation services. However, the lack of direction from municipal authorities enabled friction and disputes over available resources (e.g. land). As a result, the settlement agency, which offers more intensive services and has often limited capacity, was left out of promising opportunities. Despite this, the settlement programme decided to build a smaller garden in their facility – depending mostly on donations and internal labour – and to expand their collaboration with the food bank, promoting participation across their programmes, including cooking classes, gardening and enrolling food-insecure newcomers in the food donation programme. These actions are exemplary of not-for-profits, which with limited resources and capacity aim to improve services through referrals and collaboration.

## Discussion

This article summarizes our experience conducting a CBPE project with immigrant communities in Southern Alberta. Our research focused on evaluating the 2021 gardening season to inform programme development. The evaluation explored participants’ motivations, benefits, challenges, needs and recommendations. Immigrants were motivated by a range of interests, including meeting people, socializing, learning about gardening in Canada, re-engaging in traditional activities, having access to fresh food and being physically active. COVID-19 protocols limited opportunities for socialization, keeping those engaged without a group relatively isolated. Newcomers and older adults experienced many barriers, including family care, language limitations and demanding schedules. Exploring participants’ motivations, expectations, needs and barriers was instrumental in adapting the programme and expanding collective action beyond the food bank garden.

As previous evidence suggests, community gardens can promote community belonging and social integration (11), where immigrants from agricultural backgrounds can reconnect with traditional practices (11,14). At the same time, gardens offer opportunities to continue learning about their host country (18). Gardens can be ‘places of attunement’ (27) where newcomers adapt to the host country’s weather, soil and biodiversity. Such conditions can assist in addressing some immigrants’ needs related to social integration and belonging. Community gardens can be places of restoration, food production and belonging, forming ‘connected ecologies’ (27) for health promotion.

Strategic collaboration with immigrant communities aligns with previous action-oriented research with minorities (4,34) and the Ottawa Charter for Health Promotion (7). Throughout the project, we engaged multiple stakeholders, including service providers, grassroots immigrant organizations and community leaders, all pivotal when engaging with immigrant communities (35). Partnering organizations could learn about integrating research and action and its potential for programme development and adaptation. Research collaborators gained experience being agents of change, promoting leadership that can translate to further community action. As a network, leaders and organizations developed a sense of solidarity, which has been suggested to be crucial when advocating for resources (36). Lastly, shared decision making over every stage of the project reinforced democratic values (37), and stakeholder engagement strengthened the quality (30) and local usefulness of our research findings (29).

We identify several limitations of the project. Firstly, the engagement in the food bank’s community garden represented a rapid solution, but the context of the programme, along with COVID-19 outdoor capacity restrictions, limited immigrants’ ownership of the garden and the capacity to advocate for their food security. Creating a new community garden was a challenge without financial, social and cultural capital. Instead, we prioritized taking action with the available resources. Secondly, in 2021, recruitment and coordination relied heavily on the lead author, and establishing relationships with local organizations was challenging. However, as the network developed, the community action expanded more sustainably. In addition, we did not perform an extensive evaluation that included the measurement of socialization, belonging and other health outcomes. The findings of the evaluation are project-specific, and the size and diversity of the sample limits transferability to similar projects in other contexts. Despite this, our evaluation enabled valuable and timely information for decision making. As continuing actions demonstrated, collaborating agencies have adapted and expanded their programmes incorporating immigrants’ needs, preferences and recommendations.

Collaborating agencies have requested access to the manuscript as evidence to support their advocacy for grants and space, mainly at the municipality level. In their vision, the participation of municipalities as community partners could reduce numerous barriers. Hosting a community garden requires ongoing support and collaboration from municipal parties in issues such as supply of water, property rental, subsidies/grants and regulations. Local organizations tend to have stronger networks and ties with municipal parties. Therefore, immigrant communities may face greater challenges sustaining a community garden when organizations are no longer able to provide support. Having a continuous collaboration with municipal parties could overcome some of these challenges and facilitate long-term sustainability.

## Conclusions

The immigrant volunteering programme at the food bank garden represented a healthy, enjoyable and safe activity. Group visits facilitated community building and learning, but socialization was not experienced equally among all participants due to COVID-19 restrictions. Socialization is essential for newcomers, immigrants with limited social networks, and older adults. Maximizing socialization and community building is an important opportunity for future programming.

Our experience supports the idea that community gardens are assets that can build community resilience and promote well-being during challenging times, including pandemics (38), hurricanes (39) and earthquakes (40). Conducting a CBPE enabled us to engage immigrant communities and local organizations in meaningful ways, building capacity and collaborating toward common goals. As a network, we prioritized action. In doing so, we provided relevant and timely feedback needed to inform programme adaptation and future developments. This feedback was used by partner organizations to extend gardening activities. This approach has the potential to catalyse sustainable community action with immigrant communities.
